# Impact of provoked stress on model-free and model-based reinforcement learning in individuals with alcohol use disorder

**DOI:** 10.1016/j.abrep.2024.100574

**Published:** 2024-11-23

**Authors:** Florent Wyckmans, Armand Chatard, Charles Kornreich, Damien Gruson, Nemat Jaafari, Xavier Noël

**Affiliations:** a Laboratoire de Psychologie Médicale et d’Addictologie, Université Libre de Bruxelles (ULB), place Van Gehuchten 4, 1020 Brussels, Belgium; b Faculty of Psychology, Université de Poitiers, MSHS Bat A5 - 5, rue Théodore Lefebvre, 86073 Poitiers, France; c Cliniques Universitaires St-Luc, Av. Hippocrate 10, 1200 Brussels, Belgium; d Centre Hospitalier Henri Laborit, 370 Avenue Jacques Cœur, Pavillon Toulouse, Université de Poitiers, France

**Keywords:** Alcohol Use Disorder, Reinforcement Learning, Model-Based, Model-Free, Stress, Cortisol

## Abstract

**Background:**

From both clinical and theoretical perspectives, understanding the functionality of evaluative reinforcement learning mechanisms (Model-Free, MF, and Model-Based, MB) under provoked stress, particularly in Alcohol Use Disorder (AUD), is crucial yet underexplored. This study aims to evaluate whether individuals with AUD who do not seek treatment show a greater tendency towards retrospective behaviors (MF) rather than prospective and deliberative simulations (MB) compared to controls. Additionally, it examines the impact of induced social stress on these decision-making processes.

**Methods:**

A cohort comprising 117 participants, including 55 individuals with AUD and 62 controls, was examined. Acute social stress was induced through the socially evaluated cold pressor task (SECPT), followed by engagement in a Two-Step Markov task to assess MB and MF learning tendencies. We measured hypothalamic–pituitary–adrenal axis stress response using salivary cortisol levels.

**Results:**

Both groups showed similar baseline cortisol levels and responses to the SECPT. Our findings indicate that participants with AUD exhibit a reduced reliance on MB strategies compared to those without AUD. Furthermore, stress decreases reliance on MB strategies in healthy participants, but this effect is not observed in those with AUD.

**Conclusion:**

An atypical pattern of stress modulation impacting the balance between MB and MF reinforcement learning was identified in individuals with AUD who are not seeking treatment. Potential explanations for these findings and their clinical implications are explored.

## Highlights

• The role of Model-Free (MF) and Model-Based (MB) learning under stress in AUD is underexplored.
• This study examines how social stress affects decision-making in 55 AUD patients vs. 62 controls.
• Coordination between MB and MF learning was assessed using a Two-Step Markov task.
• Without stress, AUD individuals rely less on MB strategies compared to healthy controls.
• Stress reduces MB strategy use in healthy participants, but AUD individuals are less impacted.

## 1 Introduction

One of the most destructive characteristics of addiction is the apparent disregard for its harmful effects (American Psychiatric Association, 2013), which ultimately qualifies as a state of compulsive drug use (Everitt & Robbins, 2005). Various paths contribute to this persistence, encompassing cognitive, motivational, and behavioral elements (Brand, 2022, Everitt and Robbins, 2016, McNally et al., 2023, Noël et al., 2013a, Noël et al., 2013b, Redish et al., 2008, Zilverstand et al., 2018). Indeed, individuals with substance use disorder may exhibit significant differences in how they perceive the consequences of their actions, skewed value-based choices, or diminished abilities to exert control over their behaviors, all while maintaining accurate evaluation. For instance, as a result of repeated and prolonged alcohol consumption in predictable circumstances (e.g., feeling stressed, passing by a familiar pub), consuming alcohol may take place with limited deliberative resources and little regard for delayed or long-term consequences (Everitt and Robbins, 2005, Everitt and Robbins, 2016, Tiffany, 1990). For computational psychiatry, which has brought hope for connecting neuroscience with clinical applications (Huys, Maia, & Frank, 2016), inefficient or biased mechanisms for maximizing reward learning could contribute to a loss of control over deeply ingrained behaviors such as prolonged alcohol misuse (Groman et al., 2022, Gueguen et al., 2021, Redish et al., 2008, Sebold et al., 2022). In this study, we employed a computational approach to investigate several fundamental learning mechanisms in Alcohol-Use Disorder (AUD) and the impact of induced stress.

Essentially, the reinforcement learning (RL) theory elaborates on the need to decide between two types of anticipations of future results − experiential learning and inferential thinking (Kahnt, 2023). Experiential learning, also named Model-Free (MF) control of decision-making, involves forming associations between decisions and their outcomes over time, enabling efficient decision-making based on recovered memories of option values (Daw, Niv, & Dayan, 2005). Yet, this type of learning needs meticulous updating when results differ from expectations. Conversely, inferential thinking (or model-based – MB – controller) is crucial for less frequently or indirectly experienced decisions. MB reinforcement learning involves mentally simulating associative relationships to determine values, offering more flexibility but at a more significant computational expense (Daw et al., 2005). A typical behavioral paradigm enabling the discrimination of MB from MF forms of learning through trial-by-trial choices is the Two-Step Markov task (Daw, Gershman, Seymour, Dayan, & Dolan, 2011). In this task, MB learning refers to a ‘world model’ that includes the states, the transition probabilities between the states, and the reward of each state. MB iteratively simulates possible actions and their potential future outcomes using a learned representation called ‘model’. On the other hand, a simplified algorithm named MF can be effectively used to avoid laborious mental simulations by instead using the endpoint of all this computation, the long-run expected value of each action. Learning occurs through trial-and-error interaction with the environment every time an action is performed. Still, updating choice value is slower under MF than MB strategy.

Within this scope, a plausible vulnerability factor to compulsivity could be specific disturbances within MF and MB (RL) systems and their optimal orchestration (Gillan et al., 2016, Groman et al., 2019, Hales et al., 2023, Mollick and Kober, 2020, Sebold et al., 2022, Sharp et al., 2023, Voon et al., 2015, Wyckmans et al., 2019). Our focus is on AUD, as the existing data for this condition requires a refined interpretation (Sebold et al., 2022). Indeed, an initial study has shown that individuals with severe AUD who have recently been detoxified from alcohol display less evidence of making choices informed by MB learning than their controls (Sebold et al., 2014). However, in another study, there was no noticeable difference in the orchestration of MF/MB between the AUD group and the control group (Voon et al., 2015). Yet, it is significant that a higher reliance on MF learning may negatively impact future AUD and increase the risk of relapse. Indeed, a 3-year longitudinal study showed that MB and MF control predict different aspects of alcohol consumption in youth (Chen et al., 2021). In addition, binge drinking might also sway the MB/MF balance as the time passed since the last drinking binge impacted the MF scores, with binge drinkers showing less habitual behavior after more extended periods of abstinence (Doñamayor, Strelchuk, Baek, Banca, & Voon, 2018). This observation aligns with a substantial online study, which revealed a negative correlation between scores on the Alcohol Use Disorder Identification Test (AUDIT) and MB behavior (Gillan et al., 2016).

Regarding the risk of relapse in alcohol dependence, a complex pattern emerged, linking expectancies with reinforcement learning strategies. Specifically, individuals with high alcohol expectancies showed lower model-based control in relapsers, whereas the opposite pattern was observed in abstainers (Sebold et al., 2017). The immediate effects of alcohol could also play a crucial role in understanding the association between alcohol use and RL characteristics. For instance, intravenous ethanol infusion administered to adolescent intermediate-risk drinkers altered the balance between MB and MF learning, reducing MB choices in low-risk teenagers and enhancing this form of highly demanding goal-directed process in high-risk subjects (Obst et al., 2018). On the other hand, no correlation was found between MB, MF, and alcohol consumption in young adults who consume alcohol recreationally, that is, who did not encounter adverse consequences (Nebe et al., 2018). A key focus is understanding the RL characteristics of current drinkers with AUD who have no plans to reduce their alcohol consumption. This group comprises 90 % of Americans meeting the DSM-5 criteria for AUD (Substance Abuse and Mental Health Services Administration, 2018). Investigating RL in this context could provide insights into the persistence of harmful, habitual behaviors (Story, Vlaev, Seymour, Darzi, & Dolan, 2014).

The second claim of our research is that for a better understanding of the effects of RL mechanisms on choices made by participants with AUD in real-life situations, it's crucial to delve deeper into the impacts of affective states on these systems. Indeed, this group has shown increased alcohol consumption in response to stress (Mereish, Kuerbis, & Morgenstern, 2018), which can intensify adverse daily outcomes and lead to alcohol lapses and relapses (Sinha, 2001). An increasing body of evidence indicates that challenging operational circumstances, such as stressful events, interference, or time limitations, can potentially promote the prevalence of ingrained, overlearned responses (Dias-Ferreira et al., 2009, Otto et al., 2013, Otto et al., 2013, Radenbach et al., 2015, Schwabe and Wolf, 2010, Schwabe and Wolf, 2013, Wu et al., 2022, Wyckmans et al., 2022). For instance, when associated with low verbal working memory capacities or chronic stress, inducing cortisol salivary-stress response in healthy participants can lead to a decreased use of the MB strategy (Otto et al., 2013, Radenbach et al., 2015, Wyckmans et al., 2022) or reduced computations of both MB and MF during flexible learning (Cremer, Kalbe, Gläscher, & Schwabe, 2021). In individuals with AUD, as in other forms of addictive behaviors, it remains unclear whether stress enhances alcohol seeking by increasing craving and thereby enhancing goal-directed processes (Bresin, Mekawi, & Verona, 2018) or by fostering a more automatized form of action control generating stimuli-driven action (‘habits’; Schwabe, Dickinson, & Wolf, 2011). The first hypothesis has received support from neuroimaging studies, with stress altering the salience network by intensifying the engagement of insular regions (Bach et al., 2023, Naqvi et al., 2014). This heightened activation is associated with more robust goal-directed behaviors and increased cravings for substances. Additionally, under stress, a decision bias might engage individuals with AUD towards immediate rewards while ignoring different adverse consequences (Koob et al., 2014, Sinha, 2001). This is due to stress-related impairments in the prefrontal cortex, an essential element for cognitive control, and increased reliance on prepotent-overlearned responses. However, with countless repeated uses, drug-taking behavior can also become habitual, occurring without regard to the consequences (Everitt & Robbins, 2016). Stress also impairs the ability to learn from negative feedback (Ben Hassen, Molins, Paz, & Serrano, 2023), thus making it difficult to adjust to changing environments (including devaluation, Pritchard, Weidemann, & Hogarth, 2018), and increasing vulnerability to relapse. Together, the relationship between stress, reinforcement learning, and AUD remains hypothetical, and our study's insights could be crucial in determining whether experimentally induced stress increases the use of an MF-RL strategy or if the MB approach remains engaged in supporting goal-directed behaviors.

To assess the impact of provoked social stress on RL strategies in non-treatment seekers with an AUD, we employed a hybrid-RL 7-parameter computational model to analyze the behavioral data collected from the Two-Step Markov task (RL-task; Daw et al., 2011). We utilized the well-valid laboratory stressor named the Socially Evaluated Cold Pressor Task (SECPT) (Schwabe, Haddad, & Schachinger, 2008).

Our hypothesis posited that individuals with AUD, who are current alcohol users, would tend to repeat previously rewarded choices without thoroughly considering the task structure, thus reflecting a generalized bias towards MF learning compared to healthy controls. Given the existing disagreement within the scientific community on this subject, we refrained from making specific predictions about how stress levels might vary between individuals with AUD and controls (Chen et al., 2020). Additionally, we expect that stress responses primarily trigger a system that controls decisions based on past experiences, emphasizing cached action values (MF) (Meier et al., 2022, Otto et al., 2013).

## 2 Methods

### 2.1 Participants

Our study initially recruited 145 participants, consisting of 68 individuals diagnosed with AUD who were not seeking treatment. These diagnoses were based on a score of 16 or higher on the Alcohol Use Disorder Inventory Test (AUDIT) (Saunders, Aasland, Babor, De La Fuente, & Grant, 1993) and meeting at least two of the 11 criteria for AUD as per the DSM-5 (American Psychiatric Association, 2013). The remaining 77 participants served as healthy controls (HC). Both groups were matched for age and sex, with participation restricted to males to eliminate the need to account for the known effects of hormonal fluctuations, oral contraceptive use, and menopause on baseline cortisol levels and stress responses. It is important to note that the biological sex of all participants was fully aligned with their self-reported gender. Participants were recruited through social media advertisements and in venues where alcohol is typically consumed, primarily bars. All participants reviewed the study procedures and provided written informed consent. The CHU-Brugmann Institutional Review Board approved the study. All participants were 18 or over and underwent a comprehensive clinical diagnostic interview conducted by trained psychologists encompassing the Symptom Checklist-90-Revised (Derogatis, 1992), the Positive and Negative Affect Schedule (PANAS) (Crawford & Henry, 2004), the Beck Depression Inventory-II (Beck, Ward, Mendelson, Mock, & Erbaugh, 1961) and the State-Trait Anxiety Inventory (Spielberger, Gorsuch, Lushene, Vagg, & Jacobs, 1983). This procedure confirmed that the participants had no reported history of neurological or psychiatric diseases. Before the experiment, we conducted an alcohol breath test, excluding and rescheduling any participant who tested positive (>0g/ml). Additionally, we established success criteria for our RL-task (described at section 2.3.1.), with 10 AUD and 16 HC participants failing to meet these and consequently being excluded. Two AUD participants were further excluded due to the corruption of their cortisol samples. Thus, after carefully applying these rigorous inclusion and exclusion criteria, we obtained a final sample of 117 participants for our study.

### 2.2 Procedure

**Fig. 1:**
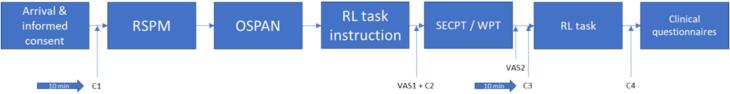
Structure of the experiment with all phases and additional measures in chronological order.

The procedure (Fig. 1) lasted 2 h and was remunerated 30€, with up to 10€ depending on task performance.

Following the completion of the informed consent form, participants engaged in the Raven’s Standard Progressive Matrices Test (RSPM) and the Operation Span Task (OSPAN). The initial cortisol measurement (C1) was obtained 10 min after their arrival. The second measurement (C2) was taken immediately after introducing the instructions for the dual-step Markov Task (RL task), along with the initial self-reported assessment (VAS1). Subsequently, the second VAS was administered immediately after the stress induction (SECPT) or control (WPT) procedure, while the third cortisol measurement (C3) was collected 10 min after the procedure. The fourth measurement (C4) was acquired immediately after completing the Two-Step Markov task. Participants concluded the session by completing various questionnaires.

First, participants were interviewed using a semi-structured format to evaluate their demographic details and drinking behaviors. Fluid intelligence was assessed with the nine-item forms of Raven’s Standard Progressive Matrices Test (see supplementary material, Bilker et al., 2012), followed by a working memory task, the Operation Span Task (OSPAN; see supplementary material, Unsworth et al., 2005), and the RL-task instructions. Before undertaking the RL-task, 50 % of the participants experienced the SECPT (Schwabe et al., 2008). In this procedure, they were recorded on video and submerged their forearm in cold water (3 °C) for three minutes under an experimenter's watchful eye. In contrast, the remaining participants went through the Warm Pressor Task (WPT), a control procedure where the water was at room temperature, and there was no observation or recording. The session concluded with participants completing clinical questionnaires. Of a total sample of 117 participants, 73 underwent the SECPT (33 HC and 40 AUD), while 44 took part in the WPT (29 HC and 15 AUD). This intentional imbalance aimed to ensure that a sufficient number of participants experienced stress, as cortisol levels were a crucial variable in our analyses. By increasing the sample size in the SECPT condition, we sought to capture a broader range of cortisol elevations, which was essential for investigating the effects of physiological stress on decision-making.

### 2.3 Measures

#### 2.3.1 Stress response assessment

Participants rated their desire to drink, feelings of stress, and pain on visual analog scales (VAS, ranging from 0 to 10) before and after the stress induction procedure. Objective stress response measures were taken in the form of salivary cortisol levels, collected 10 min after arrival (C1), immediately following the RL-task instructions (C2), 10 min post-stress induction (C3), and after the RL-task (C4). C1 and C3 were delayed by 10 min as cortisol levels are expected to peak 10 min after stress onset (McRae et al., 2006). The salivary cortisol analysis procedure is described in the supplementary material.

#### 2.3.2 Two-Step Markov task

**Fig. 2:**
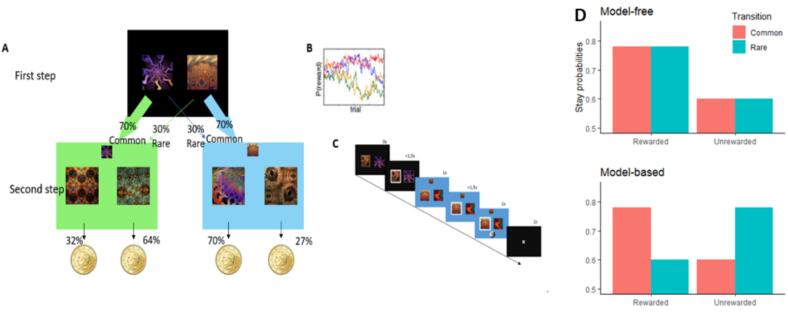
State transition and reward structure in the two-step RL task*.*

All participants were given thorough instructions for the Two-Step Markov task (RL-task; Daw et al. 2011) (see Fig. 2).

After the practice phase of 10 trial runs, they answered three pertinent questions to verify their grasp of the instructions. If any responses were incorrect, the instructions were repeated until comprehension was achieved. Subsequently, participants carried out 200 trials of the RL-task. In the initial step, they were tasked with selecting between two fractal images presented side by side on a black backdrop. Each image had a common (70 %) or infrequent (30 %) association with one of two second-step stages (as depicted in Fig. 2A). Participants were informed that these probabilities would remain constant. In the second step, subjects chose between the two images linked to probabilities to win money. Those probabilities slowly changed depending on the background color (Fig. 2B). An example of the trial’s design is presented in Fig. 2C. Fig. 2D depicts the theoretical decision pattern using pure MF and MB strategies.

Moving on to the second step, participants had to decide between two fractal images displayed side by side on a background of either green or blue, based on their choice in the first step. The probabilities of receiving rewards for each fractal image varied according to the background color. Participants were informed that these second-step probabilities would gradually fluctuate over time (following a Gaussian Random Walk with upper and lower boundaries set at 0.25 and 0.75, standard deviation = 0.025, as illustrated in Fig. 2B) to encourage ongoing exploration. After the second step, a feedback slide was presented for 1 s, indicating whether the trial resulted in a reward (a 20c coin) or no reward (“0″). Participants had 3 s to express their preference by pressing the keys ”E“ (for the left image) or ”I“ (for the right image) on an AZERTY keyboard. Each stage transition and trial interval lasted 1 s.

Before conducting data analyses, participants were automatically excluded if they failed to respond within 3 s on more than 20 occasions, consistently chose the same option in the first step in 95 % of the trials, or repeated previously rewarded second-step responses at a rate below 50 %. The MB (Model-Based) and MF (Model-Free) algorithms predict distinct observable choice patterns in subsequent two-step trials (as illustrated in Fig. 2D). Within the MF strategy, decisions are reinforced solely based on the outcomes of the prior trial. On the other hand, the choice value is updated considering the interplay between previous outcomes and transitions within the MB strategy.

### 2.4 Statistical analyses

Data cleaning and statistical analyses were performed using RStudio (v1.4.1103) and SPSS (v28). The sample size was estimated based on previous studies (Sebold et al., 2014, Voon et al., 2015). Accordingly, we aimed to find a medium-sized difference between HC and AUD with 80 % power and 95 % confidence, which required 64 participants per group.

Pre- and post-procedure cortisol concentrations were computed by averaging the first two and last two measures, respectively. Results were log-transformed because of the skewed nature of their distribution (Petzold, Plessow, Goschke, & Kirschbaum, 2010). The influence of the SECPT/WPT procedures on cortisol concentrations and subjective ratings was assessed through repeated measures MANOVA.

We fitted choice behavior on the RL-task to a 7-parameter hybrid RL algorithm (Daw et al., 2011) with the hBayesDM package (Ahn, Haines, & Zhang, 2017). Four MCMC chains of 6000 samples each were run, with the first 3000 samples as warm-up (see supplementary material). We mainly focused our analyses on the *ω*-parameter, which indicates the relative contribution of MB learning over MF learning during task completion. A multivariate regression was performed to assess the continuous effect of cortisol increase, diagnostic group, and their interaction on the *ω*-parameter.

Normality was assessed by dividing the skewness and excess kurtosis by their respective standard deviation. Distributions with both scores [-3.29; 3.29] were considered normal (Kim, 2013). The continuous variables were standardized before each regression. Demographic and clinical variables from both groups were compared with Mann-Whitney U or Welch t-tests. To avoid the confounding effect of outliers in regressions, scores that differed over three times the Median Absolute Deviation (MAD) from the median (Leys, Bernard, & Licata, 2013) were removed before each regression.

## 3 Results

### 3.1 Sample characteristics

Our ultimate sample comprised 117 individuals, 62 in the HC category and 55 in the AUD category. Table 1 illustrates the demographic and clinical attributes of both the AUD and HC groups, along with comparisons between them and Cronbach’s alpha values for each questionnaire. While AUD participants exhibited notably lower scores at the study level, these scores did not correlate significantly with our targeted computational outcomes. As a result, they were not included as covariates in our analysis models.

**Table 1:** Descriptive scores of each clinical variable and their between-subject difference.

	HC (n = 62)	AUD (n = 55)	Test	alpha
Age	30.82 (9.71) | Med = 28	32.31 (11.85) | Med = 28	U(117) = 1683, p = 0.906	N/A
Study Level	14.63 (2.35) | Med = 15	13.3 (2.98) | Med = 15	U(116) = 2104.5, p = 0.013*	N/A
AUDIT	6.58 (4.58) | Med = 5	20.69 (5.1) | Med = 19	t(109.33) = 15.66, p < 0.001*	0.88
Alcohol consumption (drink/per day)	3.39 (2.67)	6.35 (2.29)	t(115) = 5.76, p < 0.001*	N/A
Alcohol consumption frequency (4 or more times a week, in percentage)	6.5	48.9	X^2^ = 24.82, p < 0.001*	N/A
How often do you have six or more drinks on one occasion? (weekly and daily, in percentage)	11.3	67.3	X^2^ = 44.91, p < 0.001	N/A
DSM	0.59 (0.96) | Med = 0	5.37 (2.74) | Med = 5	t(70.48) = 11.07, p < 0.001*	0.85
Smoker (Yes | No)	No (n = 42) | Yes (n = 20)	No (n = 25) | Yes (n = 30)	X2(1) = 5.04, p = 0.025*	N/A
OSPAN	0.81 (0.16) | Med = 0.85	0.76 (0.15) | Med = 0.79	U(117) = 2058, p = 0.054	N/A
Raven	5.39 (1.92) | Med = 6	5.44 (2.02) | Med = 6	t(111.77) = 0.13, p = 0.893	N/A
BDI	3.74 (4.3) | Med = 2	7.29 (6.05) | Med = 6	U(117) = 1063, p < 0.001*	0.89
PANAS Pos	35.97 (6.24) | Med = 37	33.15 (7.38) | Med = 34	t(104.4) = 2.2, p = 0.03*	0.86
PANAS Neg	18.05 (6.94) | Med = 16	20.63 (7.69) | Med = 18.5	U(116) = 1317, p = 0.048*	0.89
STAI-YA	31.21 (8.74) | Med = 29	35.42 (9.85) | Med = 34	t(108.81) = 2.43, p = 0.017*	0.90
STAI-YB	39.82 (11.03) | Med = 39	43.78 (12.19) | Med = 41	t(109.71) = 1.83, p = 0.07	0.91
SRRS	242.41 (202.09) | Med = 201	228.41 (125.64) | Med = 212.5	U(115) = 1547, p = 0.577	N/A

Clinical variables assessed alcohol use disorder symptoms (AUDIT), number of DSM items (DSM), nicotine consumption, working memory performances (OSPAN), Raven score, depressive symptoms (BDI), positive affects (PANAS pos), negative affect (PANAS neg), state-anxiety (STAI-YA), trait-anxiety (STAI-YB), adverse live events (SRRS). Groups are compared with Welsh's t-tests or Mann-Whitney U according to the distribution of their scores. Cronbach's alphas are displayed when applicable. Significant group differences are displayed with a wildcard. Med = Median.

**Fig. 3:**
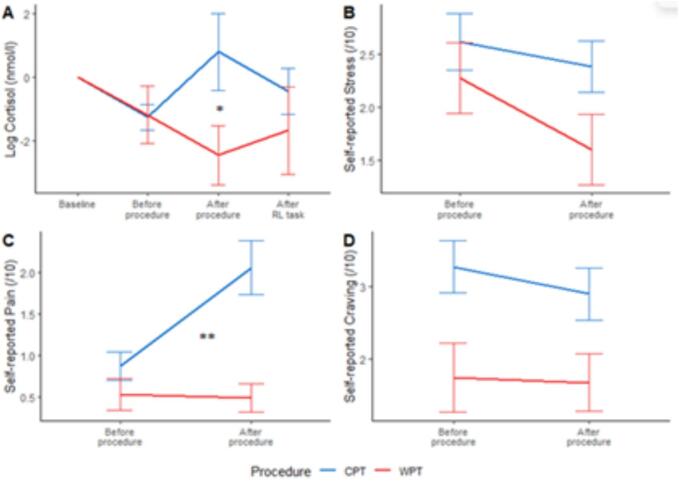
Cortisol and self-reported measures for the group that underwent the cold-pressor task (SECPT) and the Warm-Pressor Task (WPT). *Cortisol concentrations are expressed on a logarithmic scale and centered for each subject around its baseline. Graphs show mean values ± SE. P-values of the effect of interaction between time and procedure are reported.* * p < 0.05, ** p < 0.01, *** p < 0.001.

A repeated measures MANOVA was performed to evaluate the effect of stress induction procedure (between-subject; SECPT vs. WPT), time (within-subject; before vs. after the procedure), and their interaction on salivary cortisol concentrations, as well as self-reported stress, desire to drink (craving), and pain measures (Fig. 3).

The main effects of time (F(4, 112) = 4.85, p = 0.001; Wilks' Λ = 0.85, η^2^_p_ = 0.15), procedure (F(4, 112) = 2.72, p = 0.03; Wilks' Λ = 0.91, η^2^_p_ = 0.09), and their interaction (F(4, 112) = 4.43, p = 0.002; Wilks' Λ = 0.86, η^2^_p_ = 0.14) on the combined dependent variables were significant. Univariate analyses showed that participants subjected to the SECPT exhibited a more pronounced rise in cortisol levels and subjective pain ratings than in the WPT (Table 2). No group effect (AUD vs. HC) was found on pre- to post-procedure differences in cortisol concentration and subjective ratings (F(4, 112) = 1.48, p = 0.21; Wilks' Λ = 0.95, η^2^_p_ = 0.05).

**Table 2:** Cortisol Concentration and Self-Reported Measures Across Cold Pressor and Warm Pressor Tasks: Comparison Between Alcohol Use Disorder (AUD) and Healthy Control (HC) Groups.

		Cold Pressor Task (CPT)				Warm Pressor Task (WPT)				MANOVA results
Measure	Group	T1 (pre-CPT)	T2 (pre-CPT)	T3 (post-CPT)	T4 (post-task)	T1 (pre-WPT)	T2 (pre-WPT)	T3 (post-WPT)	T4 (post-task)	Time*Procedure
Cortisol concentration (nmol/l)	AUD	6.06 (3.81)	4.70 (2.50)	7.39 (12.63)	5.46 (5.17)	10.28 (7.89)	7.47 (4.28)	6.62 (3.75)	5.87 (5.69)	F(1,115) = 5.54, p = 0.02, η^2^_p_ = 0.05
	HC	7.46 (4.79)	6.34 (5.09)	7.64 (5.96)	7.23 (6.10)	8.16 (5.57)	7.84 (7.57)	6.34 (6.01)	7.92 (10.18)	
Stress (/10)	AUD		3.10 (2.46)	2.60 (2.12)			1.73 (3.17)	1.67 (2.64)		F(1,115) = 2.14, p = 0.15, η^2^_p_ = 0.02
	HC		2.03 (2.01)	2.12 (2.01)			2.66 (3.24)	2.69 (3.09)		
Pain (/10)	AUD		0.78 (1.46)	1.75 (2.76)			0.27 (0.59)	0.80 (1.52)		F(1,115) = 10.26, p = 0.002, η^2^_p_ = 0.08
	HC		1.00 (1.48)	2.42 (2.73)			0.67 (1.49)	0.33 (0.85)		
Craving (/10)	AUD		3.28 (3.09)	2.90 (3.09)			1.73 (3.17)	1.67 (2.64)		F(1,115) = 0.16, p = 0.69, η^2^_p_ = 0.001
	HC		2.67 (2.93)	3.39 (3.17)			2.66 (3.24)	2.69 (3.09)		

### 3.2 Analyses of choice behavior

**Fig. 4:**
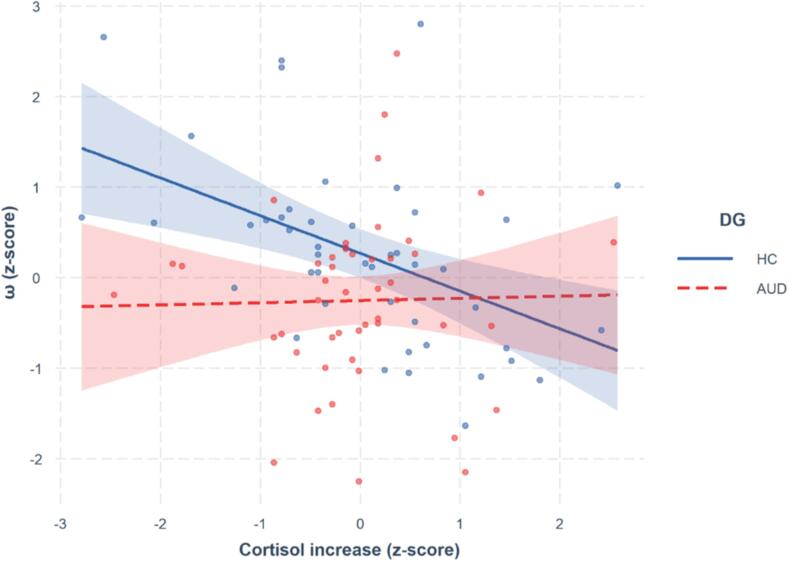
Effect of the cortisol increase on ω-parameter in AUD and HC. Each continuous score was standardized, and 95% IC is displayed around each regression line.

The ω-parameter was subjected to regression analysis involving the diagnostic group (DG; AUD vs HC), cortisol increase (dCort; continuous), and their interaction (see Fig. 4). The model demonstrated significance (F(3, 89) = 6.43, p < 0.01, R^2^ = 0.15), with notable main effects observed for DG (β = -0.52 (0.19), p = 0.01) and dCort (β = -0.42 (0.12), p < 0.001), along with a significant interaction (β = 0.44 (0.20), p = 0.03). This interaction indicated that the impact of stress on the ω-parameter was more pronounced in the HC group than in the AUD group. Bayesian Pearson’s correlations showed a negative correlation between the ω-parameter and dCort within the HC subgroup (r(46) = -0.43, BF = 52.86). In contrast, results suggested moderate evidence for the absence of a correlation within the AUD subgroup (r(46) = 0.01, BF = 0.33).

**Fig. 5:**
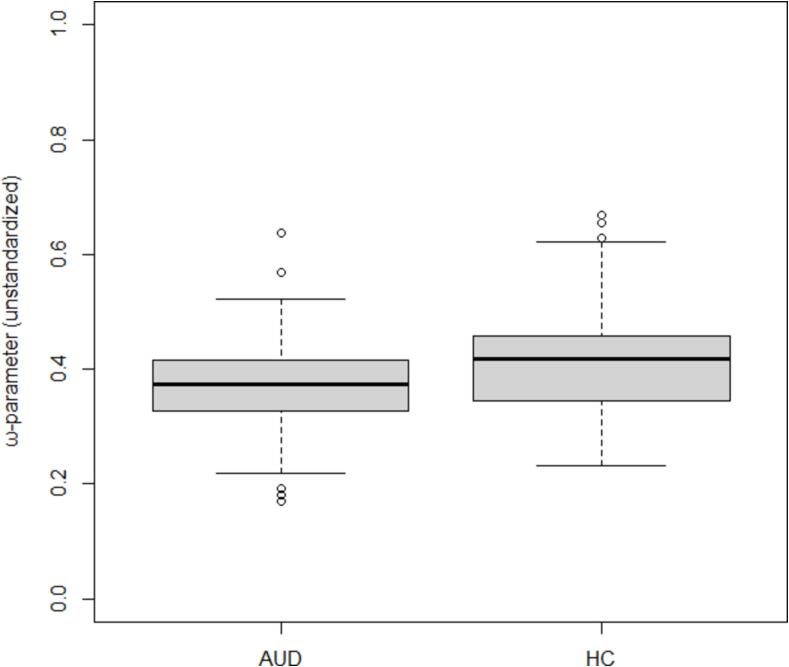
Distribution of the ω-parameter across groups.

Before conducting these analyses, 24 participants (16 HC and 8 AUD) were excluded as outliers based on one of the regression variables (see Section 2.4, Statistical analyses). Fig. 5 shows the distribution of the ω-parameter across both groups. The significance levels remained consistent, regardless of whether these outliers were included or excluded.

Considering the higher prevalence of smokers within the AUD group compared to HC, a supplementary regression analysis was executed by introducing the smoker group effect (smoker vs non-smoker). Notably, the smoker group exhibited no noteworthy main effect or interaction concerning the ω-parameter (p > 0.05), while the significance of other effects remained unchanged. Considering the marginal divergence between both groups concerning OSPAN scores, this metric was integrated into the regression model. This addition unearthed a significant main effect (β = -0.27 (0.13), p = 0.03) along with an interaction involving dCort (β = -0.20 (0.09), p = 0.04). This interaction demonstrated that higher working memory scores corresponded to elevated ω scores and mitigated the influence of cortisol elevation on the MB score. The significance of all other effects remained intact. Exploratory correlational analyses between computational measures and clinical variables are presented in supplementary information (see supplementary Fig. 2).

## 4 Discussion

The primary aim of this study was the first to explore the effect of an acute stressor on two computational systems (model-free/MF and model-based/MB) in individuals diagnosed with AUD who were actively engaged in risky alcohol consumption but were not pursuing treatment. The results revealed both groups' comparable baseline cortisol levels and stress-induced cortisol responses to a social stressor (the SECPT). Under non-stress conditions, the AUD group exhibited a reduced reliance on MB learning compared to their control counterparts. However, introducing acute stress elicits a distinct response: non-AUD participants exhibited reduced reliance on MB learning during the RL-task, whereas this pattern did not occur in the AUD group, where MB estimates remained consistent across both stressful and non-stressful conditions. Regression analyses confirmed that the stress-induced cortisol response negatively influenced the balance between MB and MF RL within healthy control subjects, a trend not mirrored in the AUD group that cannot be attributed to compromised working memory in these individuals.

The observed decrease in the use of the MB strategy during the RL-task in participants with an AUD aligns with previous research, suggesting that individuals dependent on alcohol demonstrate reduced evidence of MB strategy compared to controls (Sebold et al., 2014). However, in contrast to our findings, the between-group effects in this study did not remain significant when adjusting for cognitive capacities. In two other studies involving recently detoxified patients with severe AUD, no reduction in MB strategy was found (Sebold et al., 2017, Voon et al., 2015). As recently articulated, the inconsistent findings concerning the connection between MB behavior and AUD might be attributable to the symptom heterogeneity within AUD samples (Sebold et al., 2022). Previous research has provided evidence in support of an association between RL strategies and the risk of alcohol misuse. For instance, a negative correlation between scores on the AUDIT and MB behavior was found in a substantial study (Gillan et al., 2016). It is also significant that a higher reliance on MF learning negatively impacts future AUD (Chen et al., 2021) and a heightened relapse risk considering positive alcohol expectations (Sebold et al., 2017). The tendency towards MF strategy, observed in various studies, including our own, might be ascribed to inherent challenges in building and sustaining a thorough model of the environmental causal structure. This inclination is more than just a result of cognitive control failures, as recently suggested (Seow et al., 2021) and demonstrated in the present study.

The second main finding relates to the effect of acute stress on RL mechanisms, which differ in AUD and control groups. Our findings within the healthy control group are consistent with previous research showing that acute stress reduces MB learning (Otto et al., 2013, Wyckmans et al., 2022). More broadly, our results align with literature that indicates that demanding operational conditions—such as stressful events, interference, or constraints on time—can foster the dominance of ingrained, overlearned responses (Dias-Ferreira et al., 2009, Hardwick et al., 2019, Otto et al., 2013, Otto et al., 2013, Radenbach et al., 2015, Schwabe and Wolf, 2010, Schwabe and Wolf, 2013, Wu et al., 2022, Wyckmans et al., 2022). In contrast, participants with AUD showed no evidence that acute stress affected their MF-MB orchestration during the RL-task, unlike healthy individuals, despite having similar baseline cortisol levels and biological stress responses. The Bayesian analysis further confirmed that the increase in cortisol due to stress did not correlate with changes in MB/MF decision-making in individuals with AUD. Several potential explanations for these findings emerge.

The first is that the overall weaker verbal working memory performance in the AUD group and their usual bias towards MF learning could explain why acute stress does not detrimentally affect their MB/MF balance, a phenomenon known as the *floor effect*. Prior studies suggest that MB learning is dependent on working memory (Culbreth, Westbrook, Daw, Botvinick, & Barch, 2016), as it involves learning about action-outcome-transition connections and planning subsequent choices (Otto, Gershman, et al., 2013). Therefore, the shift from MB to MF learning among healthy individuals following stress could reduce working memory performance (Wirz, Bogdanov, & Schwabe, 2018). This implies that the cortisol surge might have a minor impact on AUD individuals with already compromised working memory, limiting the stress-driven modulation of the MB/MF balance in this group. Despite this, variations in the learning strategy modulation induced by stress between groups remained significant even after considering the effects on working memory performance. This indicates that, at most, this interpretation only partially explains our findings.

Another possible reason for the less pronounced decline in MB strategy in response to an acute stressor observed in AUD could be their higher need for MB resources to initiate goal-directed actions in response to negative emotions (Cho et al., 2019, Cooper et al., 1995). Interestingly, studies reveal that teenagers with a moderate risk of alcohol-related problems, unlike their lower-risk counterparts, demonstrated a surprising transition from MF to MB prompted by alcohol, as assessed by the RL-task (Obst et al., 2018). This outcome has been perceived as the impact of negative reinforcement swaying their decision-making. This raises the question of whether participants with AUD, when confronted with negative affects, might attempt to save their deliberative resources to support their coping mechanisms. This phenomenon could invigorate a decision-making process emphasizing addiction-related goals, serving as a response to manage and mitigate the negative affects (Hogarth, 2020). Naturally, this novel explanation invites more in-depth research to obtain a more lucid comprehension of these mechanisms.

Like any research study, our study has its limitations. Firstly, although significant, the magnitude of increased cortisol levels in response to the SECPT was less pronounced than in the initial validation study (Schwabe et al., 2008). A more unexpected finding was that the SECPT, despite eliciting a distinct painful reaction, did not increase the self-reported stress levels. This could likely be due to the participants' already relatively high baseline perceived stress levels, which averaged 2.5 out of 10. It is possible that the instruction for experimenters to remain reserved, minimize interaction, and avoid any form of reinforcement (e.g., smiling) prevented a reduction in stress during the acclimatization period. However, the elevation in salivary cortisol, which attests to a physiological response to stress, is essential for this study.

A second limitation was that our study focused exclusively on male participants. This decision was made to isolate the effects of experimentally induced stress on cortisol without the influence of other physiological factors, as the cortisol response to stress in females can vary across different phases of the menstrual cycle (Kirschbaum et al., 1992, Montero-López et al., 2018, Ozgocer et al., 2017). However, investigating RL strategies in females with AUDs is warranted, particularly considering findings demonstrating the modulation of reward-related neural function by ovarian hormones (Dreher et al., 2007).

Another potential limitation of the present study is that previous research has raised doubts about the RL-task's ability to effectively discriminate between MF and MB learning strategies (Feher da Silva & Hare, 2020). Many participants might misconceive the task in significant ways, which can impact the accuracy and reliability of the results related to the differentiation of MF and MB learning approaches. To address this limitation, we took measures to ensure that participants fully comprehended the task instructions, which were thoroughly tested in the current research.

Computational modeling offers a powerful tool for quantifying inter-individual differences in the mechanisms underlying addiction development and maintenance, holding substantial promise for promoting targeted clinical interventions (Heinz et al., 2017). For instance, participants with low MB can enhance this form of learning when higher incentives are offered (Kool et al., 2017, Patzelt et al., 2019). This enhancement can be facilitated through contingency management therapies, which involve providing tangible rewards (e.g., money) for evidence of behavioral change, such as maintaining abstinence. This type of clinical intervention has proven effective in treating substance use disorders, including AUD (e.g., Mantzari et al., 2015). Episodic future thinking training is another promising approach for reducing addiction severity (Snider, LaConte, & Bickel, 2016). For example, individuals with AUD face difficulties in vividly simulating future events (D’Argembeau et al., 2006, Noël et al., 2022), which create hyper-precise reward predictions in the habitual system and hypo-precise reward predictions in the goal-directed system (Kinley, Amlung, & Becker, 2022). Practicing episodic foresight may enhance MB control by allowing the agent to improve the precision of reward prediction in the goal-directed system (Kinley et al., 2022).

Additionally, several non-invasive brain stimulation techniques, such as transcranial direct current stimulation (tDCS) and repetitive transcranial magnetic stimulation (rTMS), may target critical neural networks involved in MB/MF orchestration (Weissengruber, Lee, O’Doherty, & Ruff, 2019). These interventions represent an emerging and promising avenue for addiction medicine (Dubuson et al., 2021, Ekhtiari et al., 2019).

The relative stability of the MB strategy in individuals with AUD, compared to those without AUD, when subjected to physical pain and elevated cortisol levels (induced by prolonged cold-water immersion) raises an important question regarding the determinants of choice and behavior in AUD under poor operating conditions. This finding sheds light on a more nuanced understanding of how stress affects clinical populations, suggesting that adversity activates goal-directed resources to cope (Hogarth, 2020). This explanation highlights the importance of clinical interventions aimed at reshaping the perceived benefits and costs of addictive behaviors, weakening outdated expectations while reinforcing updated ones. This may involve further exploration of memory reconsolidation (Elsey et al., 2018, Noël, 2023) and suppression techniques (de Almeida-Antunes et al., 2024, Noël, 2024) and extensive training to instill new expectations.

In conclusion, our study shed light on the reduced utilization of MB RL in individuals with AUD under non-stress conditions. However, the finding of resilient MB learning under acute stress provides intriguing insights into the dynamics of goal-directed control of action in this population. These results advance our understanding of reinforcement learning mechanisms under stress conditions within a population—those with AUD—where stressful episodes are known to exacerbate their condition and increase the risk of loss of control and relapse.

## CRediT authorship contribution statement

**Florent Wyckmans:** Writing – original draft, Software, Methodology, Formal analysis, Data curation, Conceptualization. **Armand Chatard:** Writing – original draft, Validation, Supervision, Conceptualization. **Charles Kornreich:** Writing – original draft, Validation, Supervision, Conceptualization. **Damien Gruson:** Writing – original draft, Investigation, Data curation, Conceptualization. **Nemat Jaafari:** Writing – original draft, Supervision, Methodology, Conceptualization. **Xavier Noël:** Writing – original draft, Visualization, Validation, Supervision, Software, Resources, Project administration, Methodology, Investigation, Funding acquisition, Formal analysis, Data curation, Conceptualization.

## Declaration of competing interest

The authors declare that they have no known competing financial interests or personal relationships that could have appeared to influence the work reported in this paper.

## Data Availability

Data will be made available on request.
